# Enlightening the Association between Bicuspid Aortic Valve and Aortopathy

**DOI:** 10.3390/jcdd5020021

**Published:** 2018-04-19

**Authors:** Froso Sophocleous, Elena Giulia Milano, Giulia Pontecorboli, Pierpaolo Chivasso, Massimo Caputo, Cha Rajakaruna, Chiara Bucciarelli-Ducci, Costanza Emanueli, Giovanni Biglino

**Affiliations:** 1 Bristol Heart Institute, Bristol Medical School, University of Bristol, Bristol BS2 89HW, UK; fs16815@bristol.ac.uk (F.S.); milano.elenagiulia@gmail.com (E.G.M.); m.caputo@bristol.ac.uk (M.C.); cha.rajakaruna@googlemail.com (C.R.); c.bucciarelli-ducci@bristol.ac.uk (C.B.-D.); costanza.emanueli@bristol.ac.uk (C.E.); 2 Department of Medicine, Division of Cardiology, University of Verona, 37100 Verona, Italy; 3 Structural Interventional Cardiology Division, Department of Experimental and Clinical Medicine, University of Florence, 50100 Florence, Italy; giuliaponte@hotmail.com; 4 Cardiac Surgery, University Hospitals Bristol, NHS Foundation Trust, Bristol BS2 8HW, UK; pierpaolo.chivasso@uhbristol.nhs.uk; 5 National Heart and Lung Institute, Imperial College London, London SW7 2AZ, UK; 6 Cardiorespiratory Unit, Great Ormond Street Hospital for Children, NHS Foundation Trust, London WC1N 3JH, UK

**Keywords:** bicuspid aortic valve, aortopathy, molecular pathways, hemodynamics, clinical management, microRNAs

## Abstract

Bicuspid aortic valve (BAV) patients have an increased incidence of developing aortic dilation. Despite its importance, the pathogenesis of aortopathy in BAV is still largely undetermined. Nowadays, intense focus falls both on BAV morphology and progression of valvular dysfunction and on the development of aortic dilation. However, less is known about the relationship between aortic valve morphology and aortic dilation. A better understanding of the molecular pathways involved in the homeostasis of the aortic wall, including the extracellular matrix, the plasticity of the vascular smooth cells, TGFβ signaling, and epigenetic dysregulation, is key to enlighten the mechanisms underpinning BAV-aortopathy development and progression. To date, there are two main theories on this subject, i.e., the *genetic* and the *hemodynamic* theory, with an ongoing debate over the pathogenesis of BAV-aortopathy. Furthermore, the lack of early detection biomarkers leads to challenges in the management of patients affected by BAV-aortopathy. Here, we critically review the current knowledge on the driving mechanisms of BAV-aortopathy together with the current clinical management and lack of available biomarkers allowing for early detection and better treatment optimization.

## 1. Introduction

Bicuspid aortic valve (BAV) is a congenital and highly heterogeneous disorder characterised by aortic valve malformations associated with aortopathy, other congenital heart defects, and genetic syndromes [1,2]. High intervention rates due to aortic valve and ascending aortic complications are observed in more than 35% of individuals born with BAV [3,4]. Despite it being the most prevalent congenital heart defect, affecting over 1% of the population [5], there are gaps in our current knowledge of different aspects of this pathology, which indeed is significantly more complex than “just” a disorder of valvulogenesis [6]. This review focuses specifically on the association between BAV and the development of aortic dilation, which is the key feature of bicuspid aortopathy [7]. The dilation can involve the ascending aorta and/or the aortic root or aortic arch [8], and BAV patients are at risk of developing thoracic aortic aneurysm (TAA) at least 10–15 years earlier than patients with a tricuspid aortic valve (TAV) [9,10]. The aim of this review is to discuss genetic and hemodynamic factors leading to or associated with BAV aortopathy and considering potential biomarkers in the light of current BAV patients’ management. There is a need to predict disease onset and elucidate its progression, thus potentially guiding the choice of optimal treatment strategies and timing [4,11,12]. Such a need is reflected by the importance of better stratifying patients, identifying peripheral biomarkers of aneurysm susceptibility, and better understanding the potential interplay between genetic and hemodynamic factors involved in the process of dilation.

Please refer to Table 1 for a list of abbreviations used throughout the article.

## 2. Tissue Biology

### 2.1. Distinct Genetic Aetiologies or Common Embryological Origin?

Linkage analyses of BAV pedigrees showed significant genetic associations, such as those located on chromosomes 5q15–21, 9q22.33 *(TGFBR1)*, 3p22 *(TGFBR2)*, 9q34–35 *(NOTCH1)*, 10q23.3 *(ACTA2)*, 13q33–qter, 15q25–q26.1, 17q24 *(KCNJ2),* and 18q, but there is not a single-gene model to explain BAV inheritance yet [13]. According to several genetic and familial clustering studies, BAV is inherited in an autosomal dominant pattern, with increasing prevalence among first-degree relatives (9%) and almost three times higher prevalence in families with more than one affected individual [4,14]. Non-valvular complications, such as TAA, are found in 10% of family members [1]. In the general population, regardless of valve morphology, 20% of the individuals with no genetic syndrome (e.g., Marfan, Ehlers Danlos, Loeys-Dietz) have a family history of thoracic aortic disease [15]. Although ascending aorta (AAo) dilation is heritable with a higher prevalence between BAV family members, it is also heritable between TAV family members, suggesting that BAV-aortopathy and valve defect may have separate genetic aetiologies. In support of the latter, an echocardiography study of 209 families enriched for BAV showed significant heritability of increasing aortic diameters, with BAV acting as an independent predictor [16]. This sets the question whether TAA is an associated, secondary, phenomenon of BAV, or whether BAV and its associated aortopathy have an oligogenic inheritance pattern, where at least two co-segregated genetic mutations are required for the development of the disease [4]. 

Familial TAAs are clinically characterized as *syndromic* or *non-syndromic*, where abnormalities in syndromic TAA go beyond the cardiovascular system [7]. Whether syndromic or non-syndromic, the association between BAV and aneurysms could be based on cell embryologic patterning [17,18]. Genetic mutations link to BAV (i.e., Nkx2.5, Alk2, eNOS, GATA5, NOTCH, Fgf8, Rock1,2, and Pax3), either in mice or human, can be subdivided in either second heart field- or neural crest cell-related. Both neural crest cells and second heart field cells contribute to semilunar valve formation [19]. Also, neural crest cell- and second heart field cell-derived SMCs populate the media of the AAo with differential distribution [20]. This suggests a common embryological pattern between BAV and its associated aortopathy. In a try to map the anatomical boundaries of second heart field cells, experiments on Nkx2-5 lineage tracing showed that second heart field cell-derived SMCs meet neural crest cell-derived-SMCs at the base of the aorta [21], and this should be further examined to better understand the associated pathologies.

Because of the evidence of a common origin between the aortic valve and the ascending aorta, it is reasonable, in fact, that genes responsible for BAV are also involved in BAV-aortopathy. Considering that the aortic root, AAo, and aortic arch derive from neural crest cells, whereas the descending aorta derives from paraxial mesoderm, a study tested the hypothesis that defective differentiation of neural crest cells-derived vascular smooth muscle cells (SMC) but not of paraxial mesoderm cells-derived SMCs contribute to BAV-aortopathy. In this study, induced pluripotent stem cells were generated from patients’ white blood cells and reprogrammed into neural or paraxial stem cells and then into SMCs. This study suggested that SMCs derived from neural crest cells in BAV-TAA subjects had impaired contractile function, decreased transforming growth factor (TGF)-β signalling, and increased mTOR signalling, unlike those derived from paraxial mesoderm cells [22].

There are two important genes that have been discussed to cause BAV. Mutations of the *NOTCH1* gene have been shown to cause abnormal migration of neural crest cells, and several studies have indicated its role in BAV disease [23,24,25,26]. Similar to *NOTCH1*, *GATA5* is known to play a role in aortic valve development and has been discussed to cause BAV [24]. Considering the shared embryological origin of the aortic valve and AAo and in light of the fact that both these genes are associated with outflow tract formation as well as valve development, it may be suggested that they are also, to some extent, implicated in the development of aortopathy in BAV patients [27]. In fact, non-synonymous variants of *NOTCH1* and *GATA5* have been associated with the coexistence of BAV and BAV-aortopathy in sporadic cases. One study observed that *GATA5*-depleted mice with BAV had also a decrease in NOTCH1 signalling pathway [28]. However, the molecular mechanisms by which those variants result in BAV and BAV-TAA are still unknown [29]. Further studies are needed to explore these mechanisms and to clarify whether BAV and its associated aortopathy arise from distinct genetic events or are instead potentially linked.

Finally, the role of eNOS during cardiovascular development was investigated through an eNOS knockout mouse model, showing association with BAV [30]. Polymorphisms in eNOS, angiotensin-converting enzyme (ACE), and matrix metalloproteinase (MMP)-2 and -9 genes have also been associated with increased risk of aneurysm development in BAV patients by examining tissue samples of different segments of the aorta, suggesting the importance of considering the histological features of the whole AAo and the genetic risk profile of the patient [31].

### 2.2. Histological Abnormalities

**Figure 1:**
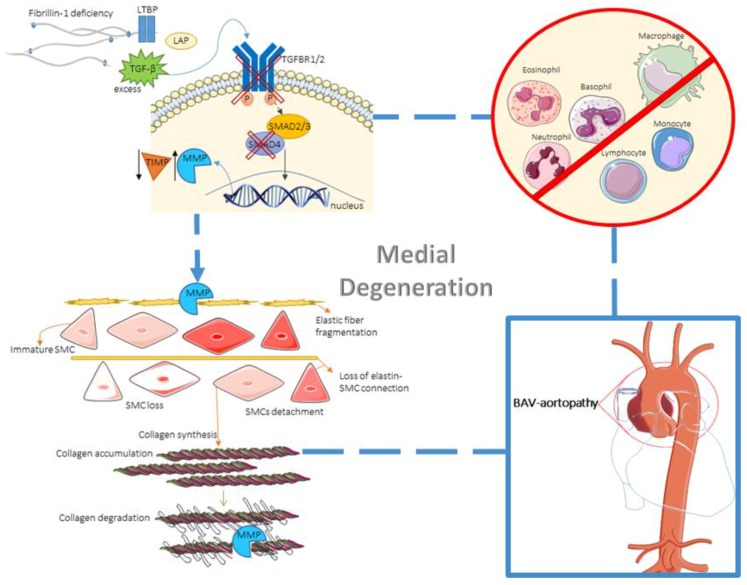
Mechanisms involved in cystic medial necrosis, eventually leading to aortopathy in BAV patients. The role of TGF-β as a mediator of ECM degradation is depicted (**top left**). Deficiency in fibrillin-1 leads to an excess of free and active TGF-β due to a decrease in microfibrils and a failure in matrix sequestration of the large latent complex (**top left**). Also, reduced microfibrils lead to decreased formation of elastic fibers. Then, TGF-β binds to its SMC membrane receptor, initiating the SMAD/TGF-β canonical pathway. Deficiency in either TGFBR1/2 or SMAD4 can also lead to aneurysm formation, along with dysregulation in the MMP/ TIMP ratio. Elastic fiber fragmentation due to MMPs takes place, as well as decrease in SMC differentiation ability and connection to elastin (**bottom left**). Collagen accumulation and eventually SMC loss and collagen degradation by MMPs can then occur (**bottom left**). All these mechanisms, in the absence of inflammation (**top right**), lead to aortopathy in BAV patients. (LAP = latency-associated peptide; P = phosphorylation).

Many studies support the presence of medial degeneration in BAV disease, characterized by: SMC loss, in the absence of inflammation; altered collagen content; elastic fiber fragmentation; accumulation of mucopolysaccharide ground substance within cell-depleted areas of the AAo media [32,33,34]. Indeed, medial degeneration (Figure 1) is considered the underling abnormality in AAo aneurysm and dissection, regardless of aetiology [32]. Considering the process of aneurysm formation in general, aortic media remodelling is characterised by altered extracellular matrix (ECM) proteins (e.g., collagen, elastin, fibrillin) production and deposition. In the context of BAV, a study of cultured BAV-SMCs obtained from aneurysmal tissue showed SMC loss in the absence of inflammation and intracellular accumulation of fibrillin, fibronectin, and tenascin, suggesting that a defect in cellular transport affects their secretion [35]. Another study on patients with BAV-TAA confirmed protein accumulation and smooth muscle cell loss and also observed elastic fiber fragmentation and decreased collagen I and III mostly in the convexity of the aorta rather than in the concavity [36]. However, it should be noted that histological research on BAV compared to TAV aortic vessel wall does not always support the increase in medial degeneration [34,37,38,39].

Ascending aortic aneurysms also exhibit elevated MMP expression. A significant increase in the MMP-2/tissue inhibitor matrix metalloproteinase (TIMP)-2 ratio has been associated with increased likelihood of BAV aneurysm formation [5,40]. Furthermore, in contrast to TAV, BAV aneurysms have been characterised by a lack of inflammation, preservation of elastin content, and increased MMP-2, implying that the pattern of MMP expression and the degree of inflammation differ between BAV and TAV scenarios, while the variations in the molecular mechanisms underlying different types of TAA need further investigation [40].

With regard to histological differences in TAA between BAV and TAV, Philippi et al. discussed that whilst TAV-TAA has a random, multidirectional collagen and elastin organisation, BAV-TAA has a highly aligned unidirectional and parallel fiber architecture, indicating that the aorta in BAV patients remodels following a unique mechanism. In addition, BAV-non-aneurysmal patients were identified to exhibit the same fiber orientation as in BAV-aortopathy patients, suggesting that such microarchitecture is a feature of BAV disease [9]. The highly aligned oriented fibers can also-at least in part-explain the relationship between BAV and increased AAo stiffness [41,42]. The same study suggests that progressive aortic dilation in BAV but not TAV patients might be associated with decreased fiber alignment as a secondary remodelling mechanism, and this could be ascribed to local changes in wall shear stress (WSS) [43,44,45].

As mentioned, SMCs play an important role in aortic aneurysm development, considering their involvement in inflammation and aortic wall homeostasis. Loss of SMC-TGFβR1 activates pathways, e.g., TGFβR2, ERK, and AngII/AT1R signals, which disrupt aortic wall homeostasis thus leading to aneurysm formation [46]. Furthermore, different molecular alterations in SMCs have been linked with aortic aneurysm formation, e.g., Smad4 or TGF-β receptor type II deficiency and subsequent increase in Cathepsin S and MMP-12, which are proteases essential for elastin degradation (Figure 1). Given the role of Smad4 as a central mediator of the canonical TFG-β signalling pathway, it has also been discussed that Smad4-deficient SMCs directly initiate aortic wall inflammation through the production of chemokines to recruit macrophages [47]. Studies have tried to unravel the role of SMCs specifically in BAV-aortopathy. Inability of differentiation in neural crest cells-derived SMCs was related to decreased expression of MYH11, caused by decreased TGF-β signalling based on the phosphorylation of SMAD2, thus indicating that decreased contractile function in these cells may contribute to the development of AAo dilation in BAV [22]. Differences in the responsiveness of SMCs to NOTCH and proosteogenic induction were identified between BAV and TAV with associated aortopathies. Osteogenic induction caused elevated RUNX2 expression in BAV patients, enhancing calcification in BAV-derived SMCs. In addition, NOTCH activation was identified to significantly increase ACTA2 expression specifically in BAV patients, leading to increased osteogenic differentiation in SMCs [48]. Finally, decreased expression of Bcl-2, a mediator of apoptosis, has been identified in BAV aortas and may be involved in SMC apoptosis [49].

In summary, despite current gaps in the knowledge of mechanisms of matrix degeneration in BAV-aortopathy, it has been suggested that SMCs in the BAV aorta exhibit an inherent defect that can lead to changes in vessel wall mechanical properties, thus contributing to aneurysm formation.

### 2.3. The Role of TGF-β

The TGF-β superfamily plays an important role in vascular remodelling and it has been the subject of extended research [50]. Dimers of mature TGF-β, as part of the small latent complex (SLC), are covalently bound to the latent TGF-β-binding protein (LTBP), resulting in the large latent complex (LLC) which then in turn binds to the ECM [51]. Fibrillin-1 is known to interact with LTBP-1 to control TFG-β activity [51,52]. A deficiency of fibrillin has been associated with BAV disease [53]. Studies comparing BAV and TAV AAo specimens identified a decrease in fibrillin-1 in BAV patients, which may contribute to explaining aortic root dilation and AAo dissection in these patients [54,55]. In two studies of BAV, TAV, and Marfan patients, both Marfan and BAV showed a differential distribution and decreased fibrillin-1 expression in the aorta, as well as significant decrease in differentiated SMC markers, suggesting a maturation defect of the aortic wall [38,39,56]. Whilst it has been suggested that deficient fibrillin-1 in BAV may lead to vascular matrix remodelling and dilatation [54], this hypothesis should be further assessed in vitro, taking into account the role of TGF-β.

Dysregulation of TGF-β, perhaps due to a faulty interaction between the SLC and the ECM, and its downstream pathways appeared to be involved in aneurysm formation [4,53]. With regards to LTBPs, these comprise a family of four ECM proteins, all having similar structure to fibrillin and, as such, possibly exerting a similar function [53], although this has not been fully demonstrated yet. One study that started to assess their function showed that LTBP4 is highly expressed in aortic ECM and interacts with matrix molecules, such as fibronectin [57,58]. Indeed, Paloschi et al. discussed an association between impaired splicing of fibronectin and increased likelihood of TAA formation in patients with BAV [59]. In addition, according to a gene expression profiling study, LTBP3 and LTBP4 were found to be highly specific for dilation in BAV rather than TAV patients [60]. The role of LTBPs and the molecules they interact with should thus be further explored in BAV-aortopathy, considering these promising initial observations.

A recent study, based on secretome analysis carried out in specimens from mildly dilated AAo with stenotic TAV or BAV and from donor normal aortas, revealed that 21 out of 38 identified dysregulated proteins were participating in TGF-β activation, emphasizing the role of TGF-β in BAV-TAA development. Decreased expression of TGFβR1 mRNA in the curvatures of BAV AAo and its positive correlation with aortic diameter suggest that TGFβR1 downregulation could be a very early event in BAV-aortopathy [61]. In addition, an altered imbalance between the TGFβR1 and the TGFβR2 subunits in BAV AAo was linked to the activation of non-canonical TGFβ-mediated signalling pathways, leading to ECM degradation and aneurysm [61,62].

Finally, a recent clinical study [63] comparing cases of TAV, BAV with dilated aorta, and BAV with non-dilated aorta indicated, by measuring a more comprehensive index of pathogenetically relevant gene expression changes, that it is not just TGF-β1 alone to play a role in this context. The ratio of circulating TGF-β1 to soluble endoglin was, in fact, found to be significantly different between TAV and BAV patients. Furthermore, this ratio was independently associated with increased MMP-2 gene expression and decreased SOD3 gene expression solely in BAV non-dilated patients, and, in this subgroup, it significantly correlated with faster aortic growth rate in the postoperative follow-up. The observed decrease in soluble endoglin in BAV non-dilated patients was also discussed as potentially being pathogenetically associated with decreased MMP-14 and endoglin gene expression in the aorta. This work can set the base for further studies.

## 3. Hemodynamics

### 3.1. Two Theories on the Pathogenesis of BAV–Aortopathy

An on-going debate exists on whether the pathogenesis of BAV–aortopathy is related to genetics or hemodynamics. A widely accepted theory (“*genetic theory*”) states that the brittleness of the aortic wall is a result of a developmental abnormality of both the aortic valve and the aortic wall. This theory is supported by the fact that BAV is a heritable defect and involves gene mutations (such as in *GATA5*, *NOTCH1* and *ACTA2* genes). A second theory (“*hemodynamic theory*”) refers to the abnormal WSS acting on the aortic wall as the process underpinning aortic dilation [64]. As it will be discussed in this section, this theory is supported by the fact that the eccentric turbulent flow through the bicuspid valve has been associated with abnormal mechanical stresses in certain regions of the aortic wall, in turn causing stress overload and wall fragility [11].

### 3.2. The Role of Hemodynamics

**Figure 2:**
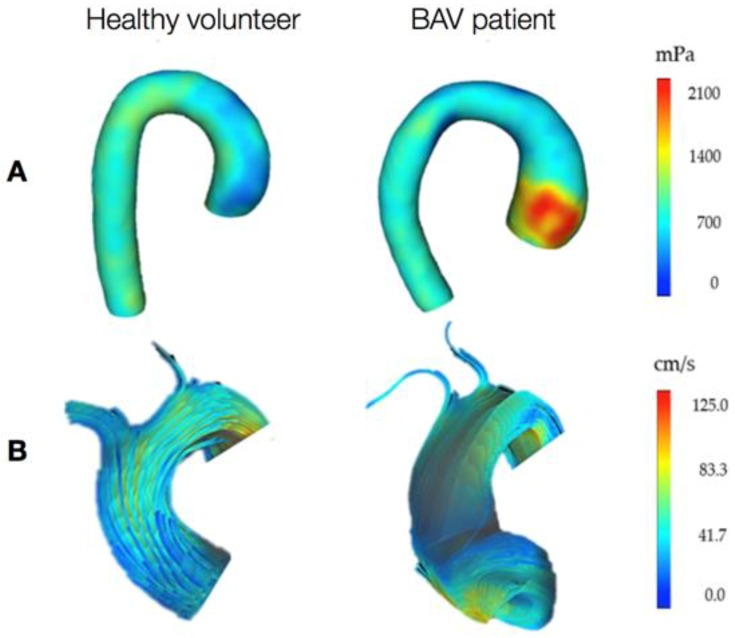
Representation of the wall shear stress (WSS) distribution (**A**, posterior view of the aorta) and flow velocity (**B**, front view of the ascending aorta) in a healthy volunteer and a BAV patient with right coronary cusp-left coronary cusp raphe, obtained from 4D flow cardiac magnetic resonance (CMR) imaging. This is a visual indication of increased WSS at the aneurysm location and abnormal flow jet in the presence of BAV. Furthermore, the flow in the BAV patient seems to go along the anterior right curvature of the AAo and then fold inferiorly along the inner curvature, rather than accessing the transverse aortic arch, as also reported in the literature [85]. Data collected at the Clinical Research and Imaging Centre (CRiC), University of Bristol; not previously published.

The turbulent flow jet passing through the BAV into the aortic root has been recognized to contribute to an abnormal biomechanical environment, including helical flow alterations that propagate eccentrically inside the proximal AAo [4]. Evidence shows that increased blood flow helicity leads to increased WSS [65], as exemplified in Figure 2. It has also been proven that the bigger the angle of the misdirected flow through the conjoined cusp opening, the higher the degree of flow eccentricity, with subsequent increased growth rate and severity of AAo dilation [66]. However, a study using convex and concave aorta biopsy samples showed different histopathological features between jet and non-jet samples [67]. This difference was similarly observed in BAV and TAV, with BAV being more significantly associated; however, this needs further assessment. In support of the hemodynamic theory, different BAV morphologies have been associated with specific dilation patterns of the AAo [68]. Some studies observed differential distributions of WSS according to different valve morphotypes, resulting in specific orientation of eccentric flow jets and suggesting possible flow-induced vascular remodelling [69,70,71]. Specifically, the most common BAV morphotype (i.e., right coronary cusp-left coronary cusp raphe) was associated with right-anteriorly directed helical systolic flow jet with peripheral skewing towards the AAo convexity, with these patients exhibiting also larger aortic root, asymmetric mid-AAo dilation, and more severe AAo wall degeneration [72,73]. On the other hand, the second commonest BAV morphotype (i.e., right coronary cusp-non-coronary cusp raphe) was associated with left-posteriorly directed eccentric flow jet spreading towards the proximal aortic arch, with patients exhibiting isolated AAo dilation, no aortic root dilation, and increased aortic arch diameter [72]. Another study confirmed that patients with right-non-coronary morphotype were more likely to present with AAo dilation, whereas patients with right-left morphotype were more likely to have aortic root dilation and, when accounting for age, they were more likely to present with aortic stenosis as well [74]. However, there are also studies reporting a weak or independent association between the BAV morphotype and the shape of the aneurysm [75,76,77]. These clinical studies were carried out on large cohorts of patients and, despite their statistical power, no association was found between morphotype and patterns of dilatation, indicating the need for further assessment of this relationship and providing evidence in support of genetic mechanisms underlying BAV-aortopathy.

Complementary to the clinical studies, experimental and computational work can also generate provocative insights into key parameters related to BAV hemodynamics. This includes systematically testing morphotype-dependent alterations and exploring whether abnormal hemodynamic parameters (such as helical flow, flow angle, or WSS) arise from the underlying abnormal valve anatomy or from the dilated aorta. One study employed particle image velocimetry to test tissue BAVs derived from porcine TAVs subjected to physiologic pulsatile flow in an experimental setup (pulse duplicator setting) and identified an element of morphotype-dependency as to which site of the aorta is affected by shear stress overloads [78]. A simulation-based study also confirmed that different BAV morphotypes affect aortic hemodynamics differently, whilst all being abnormal with respect to TAV and leading to increased WSS on the proximal AAo, and indicated BAV with left-right-coronary cusp morphotype as having the most significant abnormality [79].

The impact of altered hemodynamics on aortic wall abnormalities is also reflected in the asymmetric spatial distribution of histological and biomolecular changes in BAV aortas, in contrast to their uniform distribution in patients with Marfan syndrome [64]. According to observations by Guzzardi et al., based on 4-dimensional (4D) flow cardiac magnetic resonance (CMR) imaging data and histological analyses, increased WSS was associated with decreased elastin content and increased distance between the elastin fibers, as well as to a higher concentration of mediators of ECM dysregulation (i.e., MMPs and TGF-β) [80,81]. Regions of the dilated AAo wall with elevated WSS had significantly increased TGF-β1 concentrations [80,82]. These regions also had increased MMP-1, MMP-2, MMP-3, and higher MMP-2 to TIMP-1 activity, factors that have been associated with increased elastic fiber fragmentation [33,80]. Further studies using 4D CMR confirmed that flow asymmetry and helicity are elevated in BAV, contributing to WSS increase, and that increased WSS and medial derangement (e.g., reduced collagen and increased SMCs apoptosis) are associated with greater aortic convexity even before severe dilation is observed [5,65,73,83]. In another study based on 4D CMR, it has been hypothesised that BAV-aortopathy might be initiated by flow abnormalities and entail a sort of protective mechanism to maintain normal WSS levels in the face on the increased WSS consequent to the abnormal flow [84]. 

Experimental work also contributes to generate data in support of the effect of stresses resulting from BAV hemodynamics on aortic medial degradation. Interesting experiments have been designed by first generating values for WSS typical of both TAV and BAV hemodynamic scenarios with computational modelling, and then testing normal porcine aortic tissue in a bioreactor when subjected to such stresses and measuring relevant changes, such as MMP-2 and MMP-9 expressions, MMP-2 activity, or fibrillin-1 content. The results, however, were not conclusive [86,87], but the methodology is certainly compelling to investigate possible cause-and-effect relationships between tissue properties and hemodynamics. 

In summary, evidence exists in support of both genetic and hemodynamic theories. Clinical studies indicated that the progression of AAo dilation in BAV patients continues after aortic valve replacement leading to aortic dissection or rupture [64,88,89], which would suggest an underlying genetic driving mechanism. Other arguments in favour of the genetic theory are the enlargement of the aorta even in the absence of valvular dysfunction (i.e., stenosis and/or regurgitation) [4,5,64] as well as the fact that overall higher aortic size in BAV patients after matching for TAV patients with similar degrees of valvular disease was identified to be independent of hemodynamic alterations [64,90]. However, it should also be considered that even a functionally normal bicuspid valve is, in fact, morphologically stenotic because of the conjoined leaflets. Such valve configuration can lead to a transvalvular turbulent flow jet and abnormal haemodynamics, and jet eccentricity results in more severe flow alterations in the AAo when it occurs through a stenotic bicuspid orifice instead of a tricuspid aortic valve of comparable gradient and valve area. Furthermore, when considering cystic medial degeneration in the AAo wall of BAV patients, if, on the one hand, this phenomenon could support the genetic theory, similar changes have been identified in AAo dilation or dissection regardless of aetiology, indicating a non-specific character of cystic medial degeneration [35,91]. At present, continued research in the field and new findings from clinical, experimental, and modelling studies rather suggest an interplay between the two theories. What remains to be unravelled is which of the two can be considered as the ‘initiator’ of aortopathy, which of the two has potentially a predominant contribution, and how the interplay between the two may vary in different scenarios.

## 4. Environmental Impact

### 4.1. Environment and Risk Factors

Variable penetrance and phenotypic expression in BAV could be the result not only of genetic variations, but also of the interplay between genetic, epigenetic, and environmental modifiers, eventually leading to aortopathy. Epidemiological studies have been carried out to explore predictors of TAA in BAV patients, and their findings suggest that older age, diabetes, hypercholesterolaemia, aortic regurgitation, and smoking increase the risk of TAA [2,92]. The interplay between genetic and environmental factors has also been studied in mice [93]. It should be noted that the incorporation of multiple mutations or gene–environment interactions is required to replicate the complexity of human BAV disease [1], whereas animal models with single gene mutations may not be representative to investigate the influence of environmental hazards on complications and outcomes of the BAV disease. 

### 4.2. Epigenetic Mechanisms

The impact and interplay of environmental risk factors can also be mediated through epigenetics, and indeed studies have been performed to explore the epigenetic signature of BAV-aortopathy. 

As described in a recent study, both BAV and AAo dissection are characterized by a non-CpG hypomethylation signature that partially explains the increased cellular proliferation, although they present different DNA methylation landscapes [94]. Another study, comparing gene methylation and expression from AAo aneurysm tissue samples between BAV and TAV patients, identified hypomethylation of *ACTA2*, hypermethylation of *GATA4,* and significant hypermethylation and decreased expression of protein tyrosine phosphatase non-receptor type 22 in BAV individuals [95]. These studies suggest that altered gene methylation is involved in the pathogenesis of aneurysm formation in BAV patients. However, further investigation is needed to understand the relationships between DNA methylation and differential gene expression and whether they imply causality or are by-products of the aneurysm in the case of BAV.

The knowledge of epigenetic modifications related to TGF-β pathways in TAA could help clarify its pathogenesis. According to Kurtovic et al., a diverging alternative splicing fingerprint of the TGF-β pathways is linked to BAV-TAA, causing differential downstream effects. This might be the result of chromatin epigenetic modification, leading to BAV-aortopathy phenotypic modification [58]. In addition, induced histone methylation and acetylation of the *SMAD2* promoter in SMCs in BAV-dilated aortas was linked with SMAD2 overexpression, suggesting TGF-β/SMAD pathway dysregulation due to epigenetics [96]. The modification of histone H3 marker in the *SMAD2* promoter is an epigenetic mechanism behind SMAD2 overexpression, as demonstrated in all types of TAA, including BAV-TAA.

New insight into epigenetic reprogramming could lead to a better understanding of the onset and progression of TAA in BAV patients, thus contributing to the potential identification of novel biomarkers and/or therapeutic strategies.

## 5. The Role of MicroRNAs

MicroRNAs (miRNAs, or miRs) are small noncoding RNA regulatory molecules that regulate the expression of a plethora of mRNAs “targets”, to which they can bind, interacting with their 3′-UTR (canonical mechanism), the aminoacid coding sequence (CDS), or the 5′-UTR. The canonical mechanism consists in the miRNA seed sequence targeting one or more (semi)complementary regions of the mRNA 3′-UTR and results in expressional repression at the mRNA and/or protein level. Importantly, miRNAs can also be released by the parent cell via different shuttles, including extracellular vesicles (exosomes, microvescicles) and lipoproteins that protect their miRNA cargos from degradation. Such extracellular miRNAs can be taken up by a series of recipient cells in neighbouring and distant tissues, contributing to cell-to-cell communication. Moreover, by conferring resilience to miRNAs, the shuttles incidentally increase our possibilities to develop miRNAs as extracellular biomarkers.

This section briefly revisits the pathways so far described to affect AV morphology, BAV, and/or TAA. A number of miRNAs have been functionally implicated in processes that conduct to BAV aortopathy and/or have been found to be regulated by fluctuations in shear stress, which-as discussed-could contribute to TAA in BAV. Therefore, miRNAs could represent both therapeutic targets for intervention and circulating biomarkers that could help predict and monitor the evolution of BAV-TAA. In this setting, miRNAs regulating calcification, elastin degradation, and changes in extracellular matrix are of potential relevance. Similarly, miRNAs regulating “classic pathways” already implicated with BAV and TAA also deserve attention.

### 5.1. MicroRNAs Involved in BAV Disease and Valve Morphology

The expression and possible role of miRNAs in the calcification of stenotic BAVs has been explored in vitro using intraoperative samples in an effort to identify potentially deregulated miRNAs, as well as calcification-related factors that either regulate or are regulated by those miRNAs [97,98]. The role of the noncanonical Wnt signaling pathway [99], as well as the role of TGF-β1 in the stimulation of aortic valvular interstitial cells, leading to morphological changes consistent with myofibroblastic transformation, BMP-2 signaling, and calcification, seem to be of particular interest in relation to AV stenosis and aneurysm [100,101,102]. Since osteoblasts are involved in ECM production and mineralisation, miRNAs able to regulate osteogenesis and chondrogenesis, such as miR-29, 210, 125b, 26a, 196a, 2861, and others, are of potential relevance, even if their role in BAV is still under investigation [103,104,105,106,107,108,109,110]. Moreover, miRNAs, such as miR-29, 181a, and 195, have been shown to regulate ECM composition, acting on collagens, MMPs, and TIMPs [111,112,113]. Interestingly, miR-29b has already been shown to promote aortic valve interstitial cell calcification by inhibiting TGF-β3 through the activation of wnt3/β-catenin and to induce elastin downregulation, contributing to inorganic phosphorus-induced osteoblastic differentiation in vascular SMCs [114]. Moreover, a reduction of miR-195 in BAV was suggested to promote calcification of valve interstitial cells via SMAD7 targeting, and the mechanosensitive miR-181b regulates AV endothelial matrix degradation by targeting TIMP3 [115,116]. Other miRs reported to be deregulated in AV calcification and potentially mediating the calcification process are miR-204 and miR-449c [117,118].

### 5.2. MicroRNAs Involved in Aortopathy

Although more than 20% of TAAs are inherited as single-gene disorder, the majority are sporadic cases and known to be driven by the unbalanced production of extracellular proteases and inhibitors [119,120]. The upstream signalling events are still widely unknown; however, a potential leading cause could be altered miRNA expression, leading to gene expression impairment [120,121]. Also, it is worth mentioning that variants in miRNA genes can have a profound effect on miRNA expression and function and can thus contribute to disease [122].

Interestingly, the aetiology of descending thoracic and abdominal aortic aneurysm disease is mainly atherosclerotic, whilst proximal thoracic aortic aneurysm disease has been associated with proteoglycan accumulation, elastic fiber fragmentation, and focal or diffuse SMC degradation and loss [123]. In vitro studies using aortic samples of TAV or BAV as well as in vivo studies indicated the role of miRNAs in several pathways implicated in TAA pathogenesis, such as the focal adhesion pathway [121,124,125], ECM homeostasis [111,126,127,128], TGF-β pathway [124,129,130], and SMCs plasticity and survival [131,132].

The aforementioned miR-29, 181a, and 195 that regulate elastin and ECM could indeed be also relevant for the aneurysmal process and hence TAA [111,112,113]. Moreover, because of the fact that calcification is associated with ECM modifications, studies have been performed to appreciate the role of miRNAs with regard to elastin and ECM degradation [112,133], as well as their role on SMCs phenotypic modification, by studying Dicer-dependent miRNAs role on SMC growth, differentiation, and function in vivo [134,135,136]. 

### 5.3. MicroRNAs Regulated by Changes in Shear Stress

Vascular physiology is maintained through alterations in shear stress that potentially lead to miR-regulated differential gene expression in endothelial cells. MiRNAs induced by laminar shear stress, like miR-126, 27b, and 143/145 were identified as leading to protection from atherosclerosis, whilst miRNAs induced by low oscillatory shear stress (like miR-181b) were identified as leading to pathological vascular phenotypes [116,137,138,139], potentially triggering cardiovascular diseases.

### 5.4. MicroRNAs as Potential Therapeutic Targets and Biomarkers in BAV Aortopathy

Although most studies focus on the effect of miRNAs on either BAV morphology or aortic dilation, there are only a few that identified a relationship between *both* aortic valve morphology *and* dilation [140]. BAV patients were identified as having distinct regional miRNAs signatures in dilated aortas, indicating the differential expression of miRNAs in BAV convexity versus concavity [130]. Moreover, miRNAs were identified to influence the balance between MMP and TIMP as an early hallmark of BAV-aortopathy. Therefore, the upregulation of these miRNAs could exhibit a potential for therapeutic targeting [128]. The concept of miRNA-therapeutics essentially refers to either using “antagomiRs” or other inhibitory strategies to reduce the expression and/or activities of specific pathogenic miRNAs, or using “microRNA mimics” or other approaches to enhance the expression of a therapeutic miRNA and increase its level [141]. Compared to traditional drugs, the first generation of miRNA-therapeutics is designed to affect all the genes that are regulated by the target miRNA, thus potentially having a profound physiological impact. The potential role of miRNA therapeutics in the context of BAV-TAA, however, is yet to be explored.

In addition to their potential as therapeutic targets, those miRNAs that have been found to be functionally involved in BAV disease and morphology [142] should be further screened in BAV aortopathy (and controls) tissue and plasma/serum samples (including their extracellular vesicles components) to gain evidence of their potential as diagnostic and prognostic biomarkers.

Whilst most studies focus on analysing the expression of miRNAs in aortic tissue segments, either in patients or in animal models, few studies were carried out screening plasma of BAV-TAA patients [140,143]. Unlike tissue-specific miRNAs, circulating miRNAs can provide a complementary insight to that obtained from the complex analysis of multiple tissues [140]. They are also protected from endogenous ribonuclease-induced degradation and can be easily accessed by minimally invasive means [140,144]. Tissue or circulating miRNAs, involved in BAV morphology, aortopathy in general, or regulated by changes in shear stress, should all be specifically explored in vitro and in vivo to assess whether they play a specific role in BAV aortopathy. This could lead to the identification of new biomarkers. 

## 6. Managing BAV Aortopathy

### 6.1. Clinical Management

Despite major advances in the diagnosis, genetic screening, and ability to manipulate disease processes by pharmacological means, TAA disease can still only be managed by a combination of radiological surveillance and surgery. Presently, targets of progressive BAV disease or BAV-aortopathy are not well established, and their detection and management are still ongoing [11,145]. There is also limited understanding of the duration and long-term response to pharmacological treatments, due to the gaps in the knowledge surrounding disease pathogenesis and its underling mechanisms [11,64]. Based on the decision-making algorithm for BAV management in North America and Europe, the recommended drugs are antihypertensive agents, including β-blockers, ACE inhibitors, and angiotensin receptor blockers [146], and recent guidelines for managing AAo aneurysm in non-Marfan patients also recommend β-blockers; however, there is a lack of systematic evaluation of these agents [8,145] and in vivo studies in BAV patients [147]. Although the pharmacological treatment is debatable, it is hypothesised that blood pressure control can decrease the rate of change of central arterial pressure, leading to decreased stresses acting on the more vulnerable aneurysmal segment of the aorta, thus preventing further aortic remodelling and dilation. In theory, a reduction of aortic WSS could be an advantage of the β-adrenergic blockers, whereas the angiotensin-receptor blockers, such as Losartan, have been indicated to decrease the rate of aortic growth in patients with Marfan syndrome, but these findings should be investigated in BAV-TAA too [145,146,148]. According to McGee et al., there is no significant hemodynamic change due to medication in BAV patients [147]; however, further investigation in larger cohorts is necessary. Interestingly, the use of statins has been associated with decreased risk of clinically significant aneurysm in the abdominal aorta, which could be explained by the fact that distal aortic aneurysms are usually atherosclerotic in aetiology, and a large retrospective study reported lower odds of TAA in BAV patients taking statins preoperatively [149,150]. Nevertheless, the role of statins in this context should be explored further and prospectively before claiming a clinical benefit.

On the basis of a large survey, operative approaches and management of BAV aortopathy are quite variable and do not always follow guideline recommendations [151]. According to the guidelines (i.e., European Society of Cardiology/ESC, American Heart Association/AHA, American College of Cardiology/ACC), aortic root or AAo repair/replacement is recommended in the presence of an aortic diameter ≥ 5.5 cm in asymptomatic BAV patients, for an aortic diameter ≥ 5.0 cm in BAV patients with an additional risk for dissection, e.g., family history, or in patients at low surgical risk, and for aortic diameter ≥ 4.5 cm, in BAV patients undergoing aortic valve replacement due to severe stenosis or regurgitation [152,153,154]. Furthermore, according to the ACC and AHA guidelines, first-degree relatives of BAV patients with aortic dissection should be imaged for valvular and extravalvular complications, in order for affected individuals to receive interventions to reduce the traditional cardiovascular risk factors. Also, every newly diagnosed BAV patient is recommended to undergo a comprehensive clinical evaluation, whereas genetic tests are offered when features of single-gene disorders or syndromes are present [155]. Since BAV and syndromic TAA disease can co-exist, patients and their first-degree relatives who present with aortic dissection get offered genetic aortopathy screening.

### 6.2. Genetic Tools

The low incidence of BAV in the general population creates an obstacle for performing genome-wide association (GWA) studies. For this reason, a study analysing single nucleotide polymorphisms relevant to BAV was performed, reducing the multiple-testing penalty to control for random associations [156]. However, in order to sort through the genetic complexity of BAV–aortopathy, large patient cohorts are needed and should take into account ethnic diversity, different types of BAV, aneurysm location, and risk factors. Especially in the case of BAV, where >80% cases are sporadic, case-control association methods such as GWA studies need to be performed, requiring large samples sizes [1,157]. One recent study in a big cohort of patients (n = 466 BAV, n = 4660 controls) identified two protein-altering and regulatory genetic variants near *GATA4* [158]. Considering the existing expression and genomic data in the context of GWA studies, as well as taking advantage of system-based approaches to rank the candidate genes and screen for mutations by sequencing or copy number analysis, it is possible to achieve the identification of biologically relevant genes and pathways associated with BAV-aortopathy [156]. Causative genetic variants can be identified through family-based studies, by sequencing the entire exomes or genomes of multiple family members [159,160], whereas de novo variants, which tend to be more deleterious than inherited variations, need to be validated by performing mutation analyses [161]. At present, there are only limited reported cases of de novo variants in individuals with BAV. An example is a family with a severely affected child who received a unique variant from the mother, leading to a gain of the *PXDNL* locus on chromosome 8, whereas that particular de novo gain was also identified in the maternal uncle with known BAV [162]. The knowledge of a specific mutation in a family can enforce clinicians to screen family members and search for more clinical features. 

The discovery of new genes involved in BAV-aortopathy will lead to more targeted clinical decisions, diagnostic tests, and therapies. Since BAV–TAA displays incomplete penetrance, the associated genetic variants may also be present in unaffected individuals, indicating the need for candidate gene prioritization [163,164]. However, mutations in the same gene can lead to AAo dissection varying from recognizable genetic syndromes to sporadic diseases [165,166,167,168]. Furthermore, it is often a burden of genetic variants along with environmental factors that lead BAV patients to develop aortopathy, rather than a single variant [4,169], and, consequently, prioritizing candidate genes is more challenging. Despite the limitations of BAV animal models, targeted gene deletions in mice, zebrafish, and cultured aortic valve interstitial cells provided insight into candidate gene involvement in aortic valve development and other associated conditions [28,170,171]. High-throughput assays can be used to screen candidate genes [172]. However, there is a need for improved genetic models in order to investigate therapeutic targets. Also, “knock-in” experiments, introducing human mutations into homologous mouse or zebrafish genes, may result in models of human BAV phenotypes more promising than the current models with null mutations [173,174]. In the specific context of BAV-aortopathy, candidate genes should be investigated further, including their biological functions and the different pathways involved. Whilst these methods are beginning to be used successfully, e.g., in inherited cardiomyopathies [175,176,177], they could also be used to enlighten BAV-aortopathy pathogenesis and progression, as well as potential therapeutic solutions. 

## 7. Discussion

It is known that TAAs are associated with connective tissue diseases, BAV disease, and familial thoracic aneurysm syndrome [12]. They depict the most lethal site for aortic dilation, since they might be asymptomatic for many years and manifest as an acute rupture or dissection [123]. Considering the incidence of BAV patients who develop dilation compared to TAV patients and the lack of up-to-date knowledge in this field [5], it is important to enlighten the pathogenesis of BAV-aortopathy by stratifying the different causes and the management and research strategies, rather than discussing BAV morphology and TAA separately.

**Figure 3:**
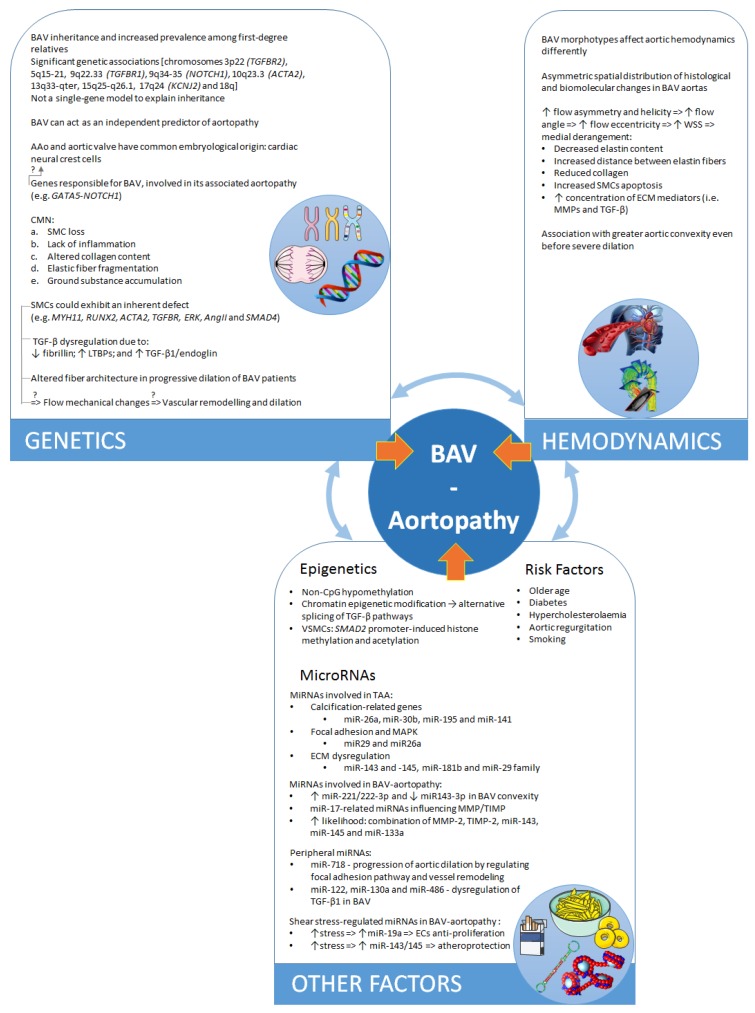
Schematic summary of the multiscale nature of the problem of BAV aortopathy and the interplay of different dimensions. The blue arrows indicate the known or possible interplay between genetics, hemodynamic alterations, and other factors underlying BAV aortopathy. These represent stimulating areas of current and future research.

Despite its clinical importance, the pathogenesis of BAV-aortopathy is widely undetermined. It is thought to involve genetic heterogeneity, ECM remodelling, abnormal signalling pathways, and aberrant neural crest cell migration, and there is evidence suggesting a distinct aetiology for BAV-TAA and the valve defect alone [33,128,178]. Nevertheless, there is also growing evidence supporting the hemodynamic derangement as a triggering and sustaining factor in BAV-TAA pathogenesis. However, it is important to emphasize that the roles of genetics and hemodynamics in BAV-TAA pathogenesis are not mutually exclusive [128] but, on the contrary, likely coexist [80], as shown in Figure 3. Indeed, BAV-TAA could be the result of a complex interaction between certain gene mutations with their modifying *loci*, hemodynamic alterations due to aberrant leaflets, and stochastic factors [179]. The disturbed balance of this interplay is likely to have an impact on disease expression and the phenotypic heterogeneity observed in BAV-TAA [4].

Population screening tools to follow TAA progression and efficient treatments to prevent the need of surgical intervention are still lacking [12,143]. Prosthetic replacement, strengthening of the aorta, and endovascular repair account for >70% of AAo procedures performed in Europe and North America and are recommended when aneurysms have reached the size or symptom thresholds for intervention [180,181,182]. There is an elevated need of effective pharmacological treatment to counterbalance and arrest aneurysmal progression and the associated increased risk of rupture [180]. The improvement of clinical decision-making and personalised care can be supported by the demonstration of cause-and-effects relationships between BAV hemodynamics and aortic wall remodelling, potentially also by using computer-based predictive models. Such models may help to predict disease onset and progression, thus potentially guiding the optimal treatment strategy [11].

Fluid dynamics has stimulated the interest of both clinicians and researchers. The assessment of aortic flow can provide disease biomarkers, particularly in BAV disease, where there is a link between hemodynamic derangement and aneurysm development. Recently, 4D CMR sequencing has allowed for macroscopic visualization and 3D quantification of thoracic aorta hemodynamics, helical flow, WSS, flow jet eccentricity, as well as the exploration of novel flow characteristics. In addition, the visualization of 3D flow features can contribute to better surgical assessment and, possibly, treatment [183]. 

Improving our understanding of the driving mechanisms of aortopathy development and progression (i.e., molecular pathways, epigenetic factors, genetic susceptibilities, and hemodynamic influences), as well as the exploration of more effective screening and therapeutic tools, are not only essential to fill the gaps mentioned above, but could also lead to identifying novel disease biomarkers and possibly also novel therapeutic targets [12]. 

How do we translate all these discoveries into clinically meaningful tools for BAV patients with aortopathy and for their families? One approach is to develop genetic tests that identify patients with BAV who are at high risk for TAA, who could then be enrolled into clinical trials to assess novel prevention strategies or drug therapies on the basis of their individualized genetic risk profiles [1]. The latter may influence surgical decisions [184]. For example, young patients with TAA with normally functioning or mildly dysfunctional BAVs frequently receive valve-sparing aortic grafts, but we do not know which of them may require future valve replacements or more distal aortic repair [185,186]. We should also consider the advantages offered by circulating miRNAs in order to identify biomarkers for the early diagnosis of BAV-TAA [128,187,188]. 

## 8. Conclusions

Whilst advances in medical imaging render our insight into aortic hemodynamics much more refined by generating not just exquisite qualitative data but also quantitative hemodynamic measurements, the link between hemodynamic derangements due to different valve morphotypes and the underlying tissue biology remains to be fully demonstrated. As reminded by important research efforts in the field [189], an association does not imply causality, nevertheless merging multimodality imaging with genotyping tools is an avenue of research that can lead to shedding light on the pathological, phenotypic, and ultimately functional aspects of this problem.

## Figures and Tables

**Table 1:** List of abbreviations.

Abbreviation	Full Name
AAo	Ascending aorta
ACE	Angiotensin-converting enzyme
BAV	Bicuspid aortic valve
ECM	Extracellular matrix
GWA	Genome wide association
LLC	Large latent complex
LTBP	Latent transforming growth factor beta binding protein
miRNA	microRNA
MMP	Matrix metalloproteinase
TAA	Thoracic aortic aneurysm
TAV	Tricuspid aortic valve
TGF	Transforming growth factor
TIMP	Tissue inhibitor matrix metalloproteinase
SLC	Small latent complex
SMC	Smooth muscle cells
WSS	Wall shear stress
